# Longevity of dental restorations in Sjogren’s disease patients using electronic dental and health record data

**DOI:** 10.1186/s12903-024-03957-9

**Published:** 2024-02-07

**Authors:** Grace Gomez Felix Gomez, Mei Wang, Zasim A. Siddiqui, Theresa Gonzalez, Oriana R. Capin, Lisa Willis, LaKeisha Boyd, George J. Eckert, Domenick T. Zero, Thankam Paul Thyvalikakath

**Affiliations:** 1 Department of Dental Public Health and Dental Informatics, Indiana University School of Dentistry, Indianapolis, IN USA; 2 Center for Biomedical Informatics (CBMI), Regenstrief Institute, Indianapolis, IN USA; 3 Department of Pharmaceutical Systems and Policy, School of Pharmacy, West Virginia University, Morgantown, WV USA; 4 Department of Biomedical Sciences and Comprehensive Care, Indiana University School of Dentistry, Indianapolis, IN USA; 5 Department of Cariology & Operative Dentistry, Indiana University School of Dentistry, Indianapolis, IN USA; 6 Department of Biostatistics and Health Data Science, Indiana University School of Medicine, Indianapolis, IN USA

**Keywords:** Direct restoration, Sjögren’s patients, Sjögren’s disease, Xerostomia, Dry mouth, Survival analysis, Dental informatics, Electronic dental record, Electronic health record

## Abstract

**Background:**

Decreased salivary secretion is not only a risk factor for carious lesions in Sjögren’s disease (SD) but also an indicator of deterioration of teeth with every restorative replacement. This study determined the longevity of direct dental restorations placed in patients with SD using matched electronic dental record (EDR) and electronic health record (EHR) data.

**Methods:**

We conducted a retrospective cohort study using EDR and EHR data of Indiana University School of Dentistry patients who have a SD diagnosis in their EHR. Treatment history of patients during 15 years with SD (cases) and their matched controls with at least one direct dental restoration were retrieved from the EDR. Descriptive statistics summarized the study population characteristics. Cox regression models with random effects analyzed differences between cases and controls for time to direct restoration failure. Further the model explored the effect of covariates such as age, sex, race, dental insurance, medical insurance, medical diagnosis, medication use, preventive dental visits per year, and the number of tooth surfaces on time to restoration failure.

**Results:**

At least one completed direct restoration was present for 102 cases and 42 controls resulting in a cohort of 144 patients’ EDR and EHR data. The cases were distributed as 21 positives, 57 negatives, and 24 uncertain cases based on clinical findings. The average age was 56, about 93% were females, 54% were White, 74% had no dental insurance, 61% had public medical insurance, < 1 preventive dental visit per year, 94% used medications and 93% had a medical diagnosis that potentially causes dry mouth within the overall study cohort. About 529 direct dental restorations were present in cases with SD and 140 restorations in corresponding controls. Hazard ratios of 2.99 (1.48–6.03; *p* = 0.002) and 3.30 (1.49–7.31, *p*-value: 0.003) showed significantly decreased time to restoration failure among cases and positive for SD cases compared to controls, respectively. Except for the number of tooth surfaces, no other covariates had a significant influence on the survival time.

**Conclusion:**

Considering the rapid failure of dental restorations, appropriate post-treatment assessment, management, and evaluation should be implemented while planning restorative dental procedures among cases with SD. Since survival time is decreased with an increase in the number of surfaces, guidelines for restorative procedures should be formulated specifically for patients with SD.

**Supplementary Information:**

The online version contains supplementary material available at 10.1186/s12903-024-03957-9.

## Introduction

Oral diseases due to salivary hypofunction constitute a significant concern among individuals with Sjögren’s disease (SD) (previously referred to as Sjögren’s Syndrome), affecting their oral and overall quality of life. The dry mouth due to salivary hypofunction is a predisposing factor for caries, candidiasis, mucositis, tooth wear, erosion, and other soft tissue oral pathologies [1, 2]. Therefore, severe dental caries and dental restoration treatments are common in this population [3]. The longevity of dental restorations is mainly affected by patient-related factors, restoration material, the tooth involved, and characteristics of the clinician who placed the initial restoration or assessed it during follow-up [4–8]. One of the principal reasons for the failure of dental restorations is recurrent caries [4, 9–11]. However, the restorative cycle of placing and replacing the restorations due to recurrent caries, fracture of the restorations, discoloration, or lack of marginal integrity leads to discomfort, pain, increasing cost, and finally, the downward spiral loss of teeth [12].

In the general population, amalgam restorations have a higher survival rate, followed by resin composites and glass ionomers [5, 13–15]. However, a recent study from one practice reported a 48% survival rate for posterior composite restorations (PCR) after 33 years and another study reported a 75% survival rate for resin composites after 15 years [16, 17]. Furthermore, systematic reviews on PCR longevity with 5–33 years of follow-up reported an overall annual failure rate of 0.08–6.3% [18–21] and, restoration survival rates ranged between 23% and 97%. Restorations failed mostly among individuals with high caries-risk, increased number of restored surfaces, and restorations affected with secondary caries [15, 22].

Individuals with SD (hereafter referred as cases) undergo extensive dental treatments due to multiple and recurrent carious lesions [23–25]. However, only a few studies have determined this patient population’s restoration longevity. These studies were either case or clinical reports or case-control studies with limited sample sizes that were insufficient to generate firm conclusions [26–30]. In addition, research on dental treatment outcomes for patients with SD is presented as anecdotal evidence gathered through surveys and interviews [23, 28, 31–33]. One recent study in Finland evaluated dental restorations among dry mouth patients, which included a subgroup of patients with SD and head and neck radiation patients showing shorter survival rates than dry mouth patients [34]. Thus, research is necessary to investigate the effectiveness of direct restorations in patients with SD to prevent tooth loss due to caries. Furthermore, no research has studied the temporal analysis of direct restorations from the time of placement at tooth surface level until replaced among this population. These types of studies need extensive data and long-term follow-up that may not be feasible through prospective clinical studies [35, 36]. However, with the increased transition of patient care documentation to electronic dental record (EDR), analyzing longevity of restorations in a larger population is possible [36, 37].

A significant barrier to studying dental treatment outcomes among patients with SD is the varying availability of medical diagnoses during dental care [38]. The historical separation of dental and medical care and the dental clinicians’ reliance on patient-reported medical conditions during dental care has led to inconsistent documentation of medical conditions in the dental patient record [39–43]. Nonetheless, the recent advances to integrate electronic dental record data with other data sources, such as electronic health record data, are improving access to patients’ medical histories for research and dental care. One previous study [44] characterized dental implant treatment survival among patients with SD using their EDR data. Thus, this study aimed to determine the longevity of direct dental restorations placed in SD patients compared to those placed in non-SD patients at the tooth and surface level using EDR and EHR data. This study is distinct because the dental patient’s SD diagnosis was established using their EHR data [38]. The findings determined the feasibility of conducting a retrospective cohort study of dental treatments among individuals with uncommon conditions, such as SD, by accessing their EDR and EHR data and the longevity of direct restoration in this population.

## Methods

### Study design, setting and patient record selection

This research is a retrospective cohort study that received exempt approval from the Indiana University Institutional Review Board (IRB approval #:1,908,582,138). In addition, a waiver of Health Insurance Portability and Accountability Act (HIPAA) authorization was obtained for collecting and using the data from health records.

**Fig. 1:**
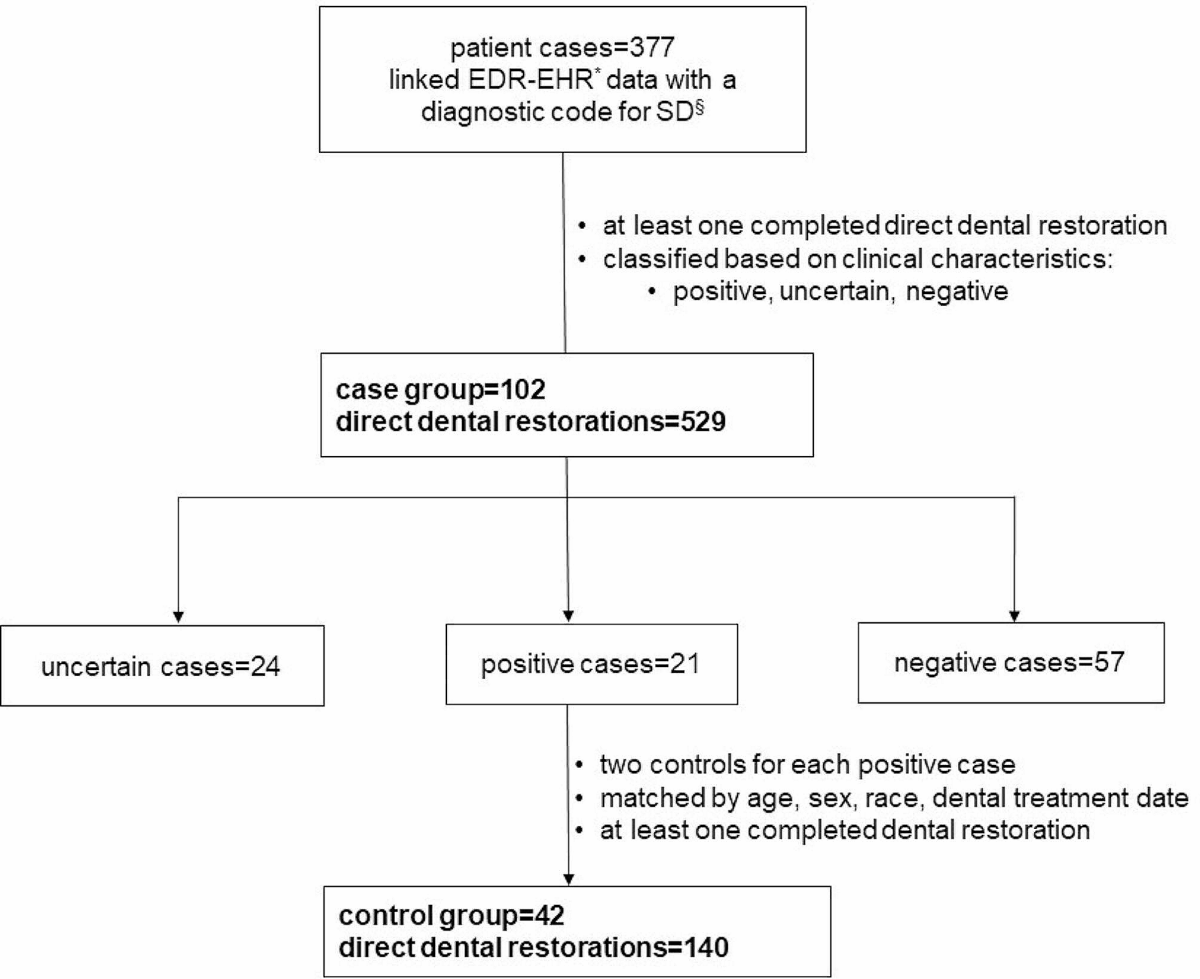
Flow diagram showing the selection case and control groups for this cohort study. The case group include patients with a linked electronic dental record-electronic health record* (EDR-EHR) data and a diagnostic code for Sjögren’s Disease^§^ (SD).

EDR (axiUm®) data of patients who received dental care in Indiana University School of Dentistry (IUSD) clinics between January 2005 and December 2020 were matched with their EHR data available through the Indiana Network for Patient Care-Research (INPC-R), maintained by Regenstrief Institute, Inc., and Indiana Health Information Exchange (IHIE), a state-wide community HIE [38, 45]. The IHIE connects 123 Indiana hospitals representing 38 hospital systems, over 19,157 practices, and 54,505 providers containing approximately 20 + million patients with more than 16 billion data elements [46–48]. 83% of IUSD patients’ EDR were matched with their EHR using the global matching algorithm [38, 49]. The final dataset included EDR data of patients 18 years and older with at least one dental restoration and SD diagnosis in their EHR (Fig. 1).

As reported previously [38] the final cohort included 441 patients with a diagnostic code for Sjögren’s Disease, ICD (International Classification of Disease)-9-CM diagnosis code: 710.2 and ICD-10-CM diagnosis code: M35.01 to M35.04, M35.09) or an internal concept code for SD (8232) used by the Regenstrief data core services (RDS). Sixty-four patient records with the following conditions that mimic symptoms and clinical characteristics of SD were excluded from the study: history of head and neck radiation treatment (including thyroid cancer patients who underwent radiation/ablation with radioactive iodine (mCi)), active hepatitis C infection, human immunodeficiency virus/acquired immunodeficiency syndrome (HIV/AIDS), sarcoidosis, pre-existing lymphoma, amyloidosis, graft versus host disease, immunoglobulin G4 (IgG4)-related disease, and primary biliary cirrhosis. The resulting 377 SD patients’ EHR data including clinical notes were manually reviewed and characterized using diagnostic guidelines based on literature review and clinical experts’ recommendations [38]. This is because the existing American European Consensus Group (AECG) criteria used to determine SD diagnosis and enroll eligible participants in clinical studies incorporate only the complete manifestation of the disease [50, 51]. As a result, physicians and rheumatologists do not use these criteria because patients present with nonspecific and heterogeneous symptoms that manifest in the early phases of SD. Thus, they were characterized as positive, uncertain, and negative SD groups [38] based on the variations in clinical findings recorded in these patients’ EHRs [38]. For instance, the patients in the negative SD group had less severe findings when compared to positive and uncertain SD groups indicating variations in disease severity in this cohort diagnosed in community practices. Moreover, healthcare providers may prefer to diagnose SD even in the absence of definitive findings so that patients benefit from symptomatic management and relief, which continue to be the primary management approach [52]. The control group included patients who did not have an SD diagnosis but had a dental treatment and matched to the characteristics of patients in the positive SD group. In addition, they were matched based on factors seen in the positive SD group that may affect dental outcomes (Fig. 1). Therefore, two control patients per positive SD case were matched by sex, age (+/- 5 years), race, and time of dental visit (+/- 3 years). Controls were at least 18 years old at the time of their dental treatment and must be completed within the study time (15 years). Controls with any exclusion conditions specified for cases and diagnostic codes for Sjögren’s were excluded. Finally, we retrieved the EDR data of SD and control group patients with at least one completed CDT (Current Dental Terminology) code for direct restorations (Additional files with supplementary Tables 1 and 2). In addition to medical insurance status, the study cohort’s systemic medical conditions (using ICD9/10-CM diagnostic codes) and medications (drug group, drug class and drug subclass through Medi-Span generic product identifier classification) that potentially cause dryness of mouth or xerostomia were retrieved from the EHR (Additional files with supplementary Tables 3 and 4). This manuscript complies with the Strengthening the Reporting of Observational Studies in Epidemiology (STROBE) guidelines [53].

### Study variables

From the EDR, the following data were retrieved: age, sex, race/ethnicity, dental insurance, completed dental treatments as CDT codes and tooth surfaces treated of eligible patients as described above (supplementary Table 1, Additional file 1; supplementary Table 2, Additional file 2). Tooth surfaces were analyzed as one, two, and three or more surfaces. From the eligible patients’ EHR, medical insurance, medical diagnosis, and medications that potentially caused dry mouth were retrieved as described above. A restorative failure was defined as the replacement of a direct restoration by any subsequent restorative dental procedure at the tooth or the same surface-level. In addition, the longevity of each direct restoration was defined as the time from the placement of the restoration to the first occurrence of a dental restoration involving the same surface of the tooth, endodontic treatment, or tooth loss. For restorations that did not fail, the last date of the CDT code of any dental treatment observed within the study period was used as the censoring time. Covariates such as presence of diagnosis for medical and other autoimmune conditions and medications used among SD cohort that potentially cause dryness of the mouth were compared with controls for restoration failure. All preventive visits were counted that included fluoride applications, caries risk assessment and management, oral hygiene instructions, periodontal maintenance, oral prophylaxis, and sealant and occlusal guard placements. The preventive visit rate per year was calculated by dividing the total number of preventive visits between the patient’s first and last dental visit of any type by the time. Similarly, the preventive visit rate per year was calculated by dividing the total number of preventive visits by the time between the first visit (of any type) and the index date for SD.

### Statistical analysis

Descriptive statistics, including frequencies, percentages, means, and standard deviations, were generated to characterize the study population. Chi-square tests or Fisher’s Exact tests were utilized to compare categorical characteristics by case/control and SD classification status, while continuous characteristics were evaluated using the independent samples t-test or Wilcoxon signed rank test and through one-way analysis of variance (ANOVA) or Kruskal-Wallis procedures. Age was calculated from date of birth to the patient’s study index date.

To compare the time to restoration failure between all case and control patients, positive SD, and control patients, and by each SD classification status, unadjusted Cox regression models were defined with random effects to account for case and control matched pairs and patients with data for multiple teeth and/or surfaces. Similarly, adjusted Cox regression models were fit to explore the effect of each of the covariates surface number, age, dental insurance, presence of diagnosis, gender, medical insurance, medication use, preventive visit rate, and race on the time to restoration failure. Kaplan - Meier plots were generated to visualize survival time by patient type and SD status.

Univariable Cox regression models without random effects were fit to examine the effects of tooth surface number, age, dental insurance, presence of diagnosis, gender, medical insurance, medication use, race, preventive visit rate per year, and SD status on time to restoration failure within case patients only. The proportional hazard odds assumption was examined by adding a time-dependent explanatory variable to the model.

A 5% significance level was used for all analyses. All statistical analyses were performed using SAS version 9.4 (SAS Institute, Inc., Cary, NC). All analytical assumptions were verified prior to analysis.

## Results

Within the 15-year study period, 102 cases and 42 matched controls (*N* = 144) were included with at least one completed direct dental restoration. The 102 cases had 529 restorations: 108 amalgam restorations, 420 resin composite restorations, along with 1 unspecified restorative procedure. Of these resin composite restorations, 247 were anterior- and 174 were posterior-composite restorations. The 42 controls had 140 restorations: 30 amalgam restorations and 110 resin composite restorations, 68 of which were anterior and 42 of which posterior composite restorations. The cases were characterized as positive, negative, and uncertain cases based on clinical findings in the EHR. The patient characteristics are listed in Table 1 for the cases and controls and Table 2 for the three SD case classifications based on clinical findings (positive, negative, and uncertain). The average age (mean ± sd) of the study cohort on their index date was 55.9 ± 13.9 years. 93% were female, about 93% had the presence of a medical condition that potentially causes dry mouth, and 94% had a record of the use of a medication that potentially causes dry mouth. 61% had public medical insurance, 74% had no dental insurance (self-paid), and 1% had public dental insurance. The mean preventive dental visit rates were 0.8 per year (range: 0.3, 1.1) between the first and last dental visit and 1 per year (range: 0.3, 2.3) between the first visit and the index date for SD. Cases and controls differed only based on the presence of a diagnosis for a medical condition that potentially causes dry mouth (*p* < 0.05) because 99% of cases had at least one medical diagnosis. Similarly, the three SD classifications and controls differed in the presence of a medical condition that causes dryness of mouth (*p* < 0.05).

**Table 1:** Summary table of patient characteristics by cases and controls (*N* = 144)

Variable	Overall *N* = 144^a^		Case *N* = 102		Control *N* = 42		*p*-value
**Age, years, mean ± SD**	55.9 ± 13.9		55.5 ± 13.9		57.0 ± 14.1		0.56
**Age group ** ***n *** **(%)**	***N***	**(%)**	***N***	**(%)**	***N***	**(%)**	
20–29	5	(3.5)	3	(2.9)	2	(4.8)	
30–39	11	(7.6)	9	(8.8)	2	(4.8)	
40–49	33	(22.9)	26	(25.5)	7	(16.7)	
50–59	30	(20.8)	19	(18.6)	11	(26.2)	
60–69	45	(31.3)	31	(30.4)	14	(33.3)	
70–79	15	(10.4)	10	(9.8)	5	(11.9)	
80+	5	(3.5)	4	(3.9)	1	(2.4)	
**Sex, ** ***n *** **(%)**							0.72
Female	134	(93.1)	94	(92.2)	40	(95.2)	
Male	10	(6.9)	8	(7.8)	2	(4.8)	
**Race, ** ***n *** **(%)**							0.61
Black or African American	21	(14.6)	13	(12.7)	8	(19.0)	
White	78	(54.2)	56	(54.9)	22	(52.4)	
Unknown	45	(31.3)	33	(32.4)	12	(28.6)	
**Ethnicity, ** ***n *** **(%)**							0.61
Hispanic or Latino	7	(5.4)	6	(6.8)	1	(2.4)	
Not Hispanic or Latino	1	(0.8)	1	(1.1)	0	(0.0)	
Unknown	122	(93.8)	81	(92.0)	41	(97.6)	
**Dental insurance, ** ***n *** **(%)**							0.88
Government	2	(1.4)	2	(2.0)	0	(0.0)	
Grant	1	(0.7)	1	(1.0)	0	(0.0)	
Private	35	(24.3)	26	(25.5)	9	(21.4)	
Self-Pay	106	(73.6)	73	(71.6)	33	(78.6)	
**Medical insurance, ** ***n *** **(%)**							0.68
Commercial	44	(32.6)	33	(35.1)	11	(26.8)	
Public	82	(60.7)	55	(58.5)	27	(65.9)	
Others	9	(6.7)	6	(6.4)	3	(7.3)	
**Medical diagnosis, ** ***n *** **(%)**							< 0.001*
Yes	133	(93.0)	101	(99.0)	32	(78.0)	
No	10	(7.0)	1	(1.0)	9	(22.0)	
**Medication use, ** ***n *** **(%)**							0.45
Yes	135	(93.8)	97	(95.1)	38	(90.5)	
No	9	(6.3)	5	(4.9)	4	(9.5)	
**Preventive visit rate** (First-Last visit)^b^, median (q1, q3)	0.8 (0.3,1.1)		0.8 (0.3,1.3)		0.7 (0.3,1.0)		0.47
**Preventive visit rate** (First visit-Index date)^c^, median (q1, q3)	1.0 (0.3, 2.3)		1.0 (0.3, 2.2)		1.2 (0.5, 2.5)		0.51

^a^The study cohort included 144 SD cases and control patients with at least one completed direct dental restoration treatment code; SD is Standard deviation; Asterisks (*) indicate statistical significance at the 5% level. ^b^The preventive visit rate per year was calculated by dividing the total number of preventive visits by the absolute value of the time, between the patient’s first dental visit and last dental visit (of any type). Absolute times values less than one year were rounded to the nearest year. ^c^ The preventive visit rate per year was calculated by dividing the total number of preventive visits by the absolute value of the time, between the index date and the patient’s first dental visit (of any type). Absolute times values less than one year were rounded to the nearest year

**Table 2:** Patient characteristics by Sjögren’s disease status (positive, uncertain, negative) and controls (*N* = 144)

Variable	Positive *N* = 21		Negative *N* = 57		Uncertain *N* = 24		Control *N* = 42		*p*-value
**Age, mean ± SD**	52.0 ± 13.4		56.5 ± 14.4		56.2 ± 13.1		57.0 ± 14.1		0.58
**Age Group**	***N***	**(%)**	***N***	**(%)**	***N***	**(%)**	***N***	**(%)**	
20–29	1	(4.8)	2	(3.5)	0	(0.0)	2	(4.8)	
30–39	3	(14.3)	4	(7.0)	2	(8.3)	2	(4.8)	
40–49	5	(23.8)	14	(24.6)	7	(29.2)	7	(16.7)	
50–59	5	(23.8)	11	(19.3)	3	(12.5)	11	(26.2)	
60–69	6	(28.6)	15	(26.3)	10	(41.7)	14	(33.3)	
70–79	1	(4.8)	8	(14.0)	1	(4.2)	5	(11.9)	
80+	0	(0.0)	3	(5.3)	1	(4.2)	1	(2.4)	
**Sex, ** ***n *** **(%)**									0.30
Female	19	(90.5)	51	(89.5)	24	100.0)	40	(95.2)	
Male	2	(9.5)	6	(10.5)	0	(0.0)	2	(4.8)	
**Race, ** ***n *** **(%)**									0.15
Black or African American	5	(23.8)	8	(14.0)	0	(0.0)	8	(19.0)	
White	10	(47.6)	28	(49.1)	18	(75.0)	22	(52.4)	
Unknown	6	(28.6)	21	(36.8)	6	(25.0)	12	(28.6)	
**Ethnicity, ** ***n *** **(%)**									0.76
Hispanic or Latino	1	(5.9)	3	(6.1)	2	(9.1)	1	(2.4)	
Not Hispanic or Latino	0	(0.0)	1	(2.0)	0	(0.0)	0	(0.0)	
Unknown	16	(94.1)	45	(91.8)	20	(90.9)	41	(97.6)	
**Dental insurance, ** ***n *** **(%)**									0.47
Government	1	(4.8)	1	(1.8)	0	(0.0)	0	(0.0)	
Grant	0	(0.0)	1	(1.8)	0	(0.0)	0	(0.0)	
Private	8	(38.1)	14	(24.6)	4	16.7)	9	(21.4)	
Self-Pay	12	(57.1)	41	(71.9)	20	83.3)	33	(78.6)	
**Medical insurance ** ***n *** **(%)**									0.93
Commercial	7	(35.0)	20	(37.0)	6	(30.0)	11	(26.8)	
Public	11	(55.0)	31	(57.4)	13	(65.0)	27	(65.9)	
Others	2	(10.0)	3	(5.6)	1	(5.0)	3	(7.3)	
**Presence of medical diagnosis ** ***n *** **(%)**									< 0.001*
Yes	21	(100.0)	56	(98.2)	24	(100)	32	(78.0)	
No	0	(0.0)	1	(1.8)	0	(0.0)	9	(22.0)	
**Medication use ** ***n *** **(%)**									0.66
Yes	21	(100.0)	53	(93.0)	23	(95.8)	38	(90.5)	
No	0	(0.0)	4	(7.0)	1	(4.2)	4	(9.5)	
**Preventive visit rate per year** (First-Last visit)^a^, median (q1, q3)	0.94 (0.32,1.53)		0.63 (0.21,1.10)		0.83 (0.43, 1.16)		0.73 (0.29,1.00)		0.551
**Preventive visit rate** per year (First visit-Index date)^b^, median (q1, q3)	1.32 (0.68, 2.23)		0.72 (0.07, 2.00)		1.00 (0.33, 4.00)		1.17 (0.50, 2.53)		0.458

The SD is Standard deviation; Asterisks (*) indicates statistical significance at the 5% level. ^a^The preventive visit rate per year was calculated by dividing the total number of preventive visits by the absolute value of the time between the patient’s first dental visit and last dental visit (of any type). Absolute times values less than one year were rounded to the nearest year. ^b^The preventive visit rate per year was calculated by dividing the total number of preventive visits by the absolute value of the time between the index date and the patient’s first dental visit (of any type). Absolute times values less than one year were rounded to the nearest year

**Fig. 2:**
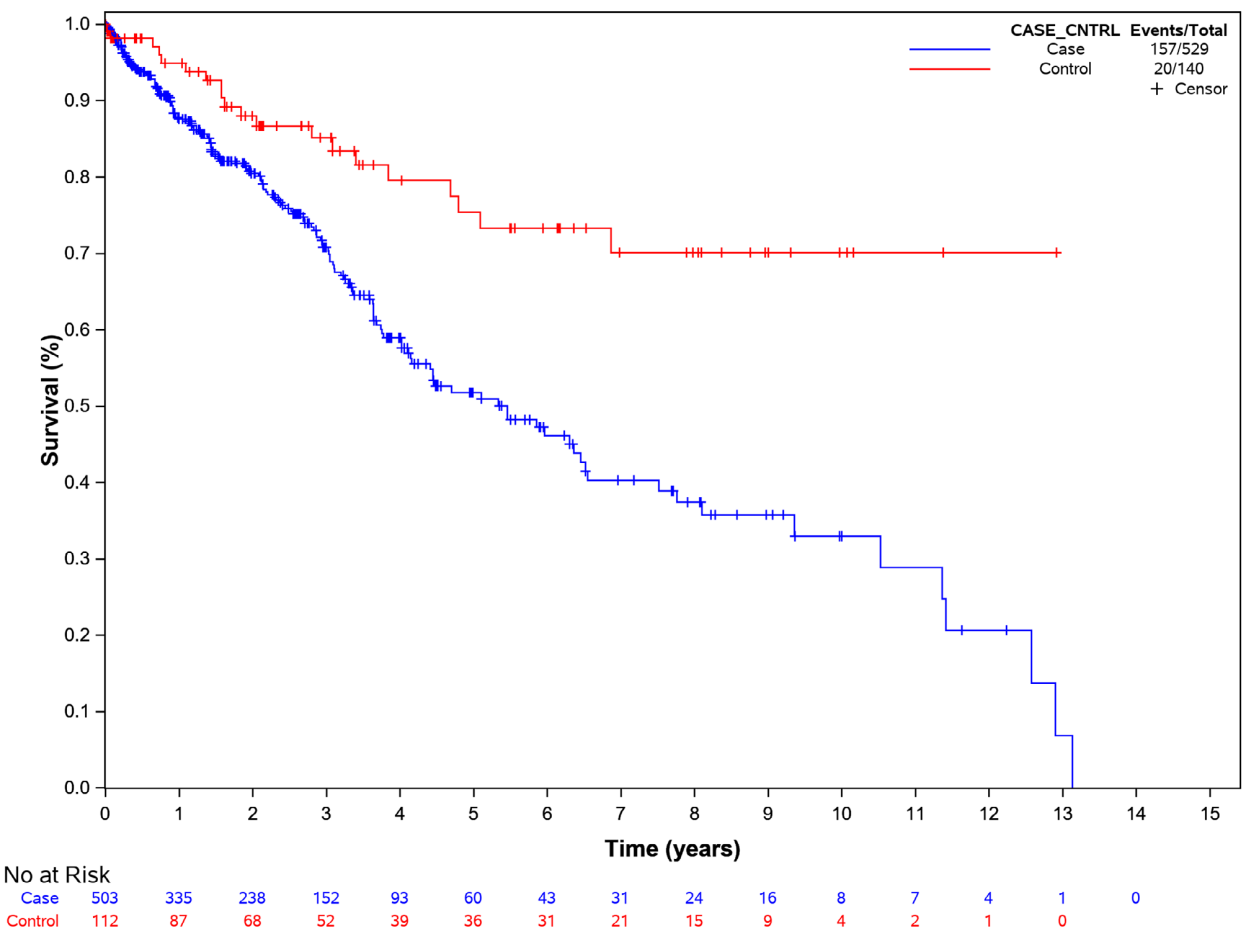
Kaplan-Meier plot for time to restoration failure by patient type with all cases (*N* = 144). Survival analysis shows higher failure rate among cases compared to controls. Hazard ratio (HR) is 2.99 with confidence interval (CI) ranging from 1.48 to 6.03. *p* value is 0.002

Matched pair univariate analysis showed that the direct dental restoration failure risk was 199% higher in cases than in non-SD controls (HR: 2.99, 95% confidence interval (CI) 1.47–6.03, *p* = 0.002, Fig. 2). After controlling for the number of tooth surfaces, cases were 2.76 times more likely to experience a restoration failure than non-SD controls (HR: 2.76 95% (1.37–5.57), *p* = 0.005). Matched pair analysis controlling for cases and controls showed that restorations with two or more surfaces failed faster than single-surface restorations (*p* < 0.05) (HR:1.74, 5% (1.115, 2.710), *p* = 0.015) (Table 3). Although the reasons for restoration failures could not be determined, the commonly performed failure procedures were restorations (direct and indirect) (74%), tooth extractions (22%) and endodontic treatments (4%). None of the other covariates included in the analysis were significant (*p* > 0.5) for all case and control comparisons. Examining the Kaplan-Meier curves (Fig. 2), at year five, the probability of survival for dental restoration was about 55% with 60 out of 529 restorations still at risk in cases and the probability of dental restoration survival was about 75% with 36 out of 140 restorations still at risk among non-SD controls.

**Table 3:** Dental restoration failure by patient type with covariates for all cases and controls (*n* = 144)

Model	Variable	Comparison	Hazard Ratio (95% CI)	*p*-value
Patient Type + Age^a^	Patient Type	Case vs. Control	2.976 (1.468, 6.033)	0.003*
	Age	Each 1-Year Change	1.005 (0.984, 1.026)	0.657
Patient Type + Sex^a^	Patient Type	Case vs. Control	3.044 (1.495, 6.198)	0.002*
	Sex	Female vs. Male	1.173 (0.492, 2.799)	0.719
Patient Type + Race^a^	Patient Type	Case vs. Control	2.906 (1.420, 5.946)	0.004*
	Race			0.346
		Black vs. White	0.679 (0.298, 1.545)	0.356
		Unknown vs. White	1.022 (0.537, 1.946)	0.947
Patient Type + Dental Insurance^a^	Patient Type	Case vs. Control	2.985 (1.477, 6.031)	0.002*
	Dental Insurance	Other vs. Self-Pay	1.021 (0.684, 1.523)	0.919
Patient Type + Medical Insurance^b^	Patient Type	Case vs. Control	2.916 (1.395, 6.094)	0.004*
	Medical Insurance			0.459
		Commercial vs. Public	1.343 (0.734, 2.457)	0.339
		Other vs. Public	2.171 (0.435, 10.829)	0.345
Patient Type + Presence of Medical Diagnosis^c^	Patient Type	Case vs. Control	3.257 (1.561, 6.800)	0.002*
	Presence of Diagnosis	Yes vs. No	0.562 (0.160, 1.980)	0.370
Patient Type + Medication Use^a^	Patient Type	Case vs. Control	3.006 (1.484, 6.089)	0.002*
	Medication Use	Yes vs. No	1.451 (0.363, 5.795)	0.598
Patient Type + Preventive Visit Rate per year^a,d^	Patient Type Preventive Visit	Case vs. Control Each 1-visit increase	2.990 (1.476, 6.061) 0.994 (0.882, 1.121)	0.002* 0.928
Patient Type + Surface Number^a^	Patient Type	Case vs. Control	2.758 (1.365, 5.571)	0.005*
	Surface Number			0.022*
		2 vs. 1	1.473 (1.014, 2.140)	0.042*
		3 + vs. 1	1.738 (1.115, 2.710)	0.015*
		3 + vs. 2	1.180 (0.731, 1.904)	0.497

^a^The study cohort for this analysis included 144 case and control patients with at least one dental restoration treatment code. The sample includes 140 restorations for control patients and 529 restorations for case patients for a total of 669 restorations. ^b^The study cohort for this analysis included 94 cases with 463 restorations and 41 controls with 139 restorations for a total of 602 restorations. ^c^The study cohort for this analysis included 102 cases with 529 restorations and 41 controls with 139 restorations for a total of 668 restorations. ^d^The preventive visit rate per year was calculated by dividing the total number of preventive visits by the absolute value of the time between the index date and the patient’s first dental visit (of any type). Absolute times values less than one year were rounded to the nearest year. Asterisks (*) indicates statistical significance at the 5% level

**Fig. 3:**
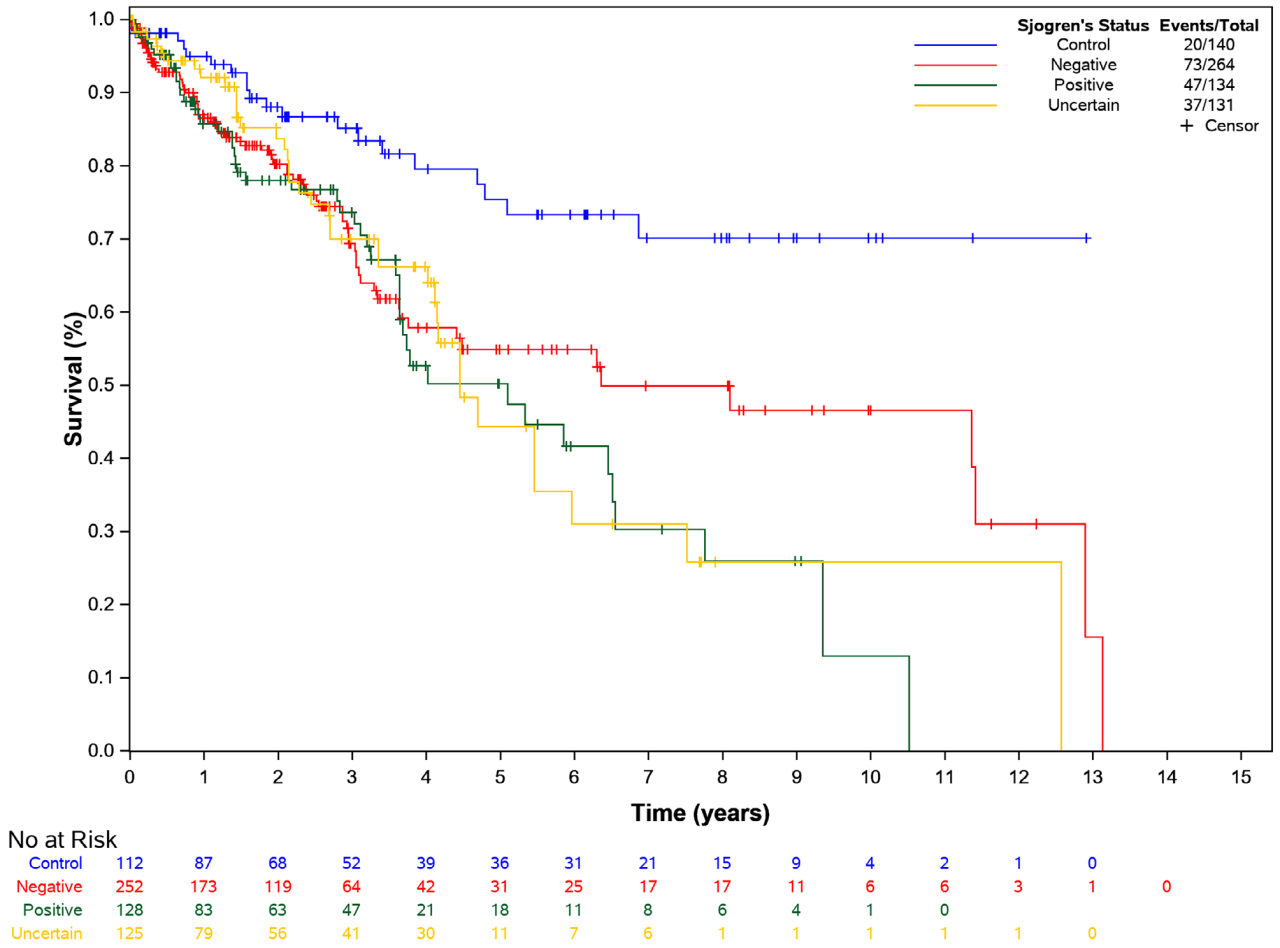
**Kaplan-Meier plot for time to restoration failure by Sjögren’s disease status.** Survival analysis shows higher failure rate among positive cases compared to controls. Hazard ratio (HR) for positive case is 3.18 with confidence interval (CI) ranging from 1.38 to 7.33; *p* value is 0.007; HR for uncertain case is 2.73 with CI ranging from 1.08–6.89; *p* value is 0.034; HR for negative case is 2.98 with CI ranging from 1.37–6.49; *p* value is 0.006

When the cases were split into positive, uncertain, and negative, similar results were found when comparing against controls within all three subgroups: HR 3.2 (1.38–7.33, *p* = 0.007) for positive, HR 2.73 (1.08–6.89, *p* = 0.034) for uncertain, HR 2.98 (1.37–6.49, *p* = 0.006) for negative (Fig. 3). Matched pair analysis controlling for positive cases and controls showed that restorations with two or more surfaces failed faster than single-surface restorations (*p* < 0.05) (Table 4). None of the other covariates included in the analysis were significant (*p* > 0.5) for all case and control comparisons. The Kaplan-Meier curves for positive, uncertain, and negatively classified SDs (Fig. 3) estimated 5-year survival was at approximately 50%, 45%, and 55%, respectively. When analyzing only the cases (excluding controls from the analysis), none of the other factors were found to be significant (*p* > 0.05) (Table 5). Because of the limited overall number of failures, missing data in some covariates, and no differences between groups for the covariates, no multivariable analysis was performed.

**Table 4:** **Matched pair analysis for time to dental restoration failure by cases and controls with covariates for only positive cases and controls (** *n* **= 63)**

Model	Variable	Comparison	Hazard Ratio (95% CI)	*p*-value
Patient Type + Age^a^	Patient Type	Case vs. Control	3.293 (1.456, 7.449)	0.004*
	Age	Each 1-Year Change	1.003 (0.968, 1.040)	0.852
Patient Type + Sex^a^	Patient Type	Case vs. Control	2.990 (1.290, 6.930)	0.011*
	Sex	Female vs. Male	0.450 (0.098, 2.072)	0.305
Patient Type + Race^a^	Patient Type	Case vs. Control	3.149 (1.405, 7.060)	0.005*
	Race			0.256
		Black vs. White	0.341 (0.086, 1.357)	0.127
		Unknown vs. White	0.609 (0.191, 1.941)	0.402
Patient Type + Dental Insurance^a^	Patient Type	Case vs. Control	3.220 (1.458, 7.112)	0.004*
	Dental Insurance	Other vs. Self-Pay	1.182 (0.675, 2.069)	0.559
Patient Type + Medical Insurance ^b^	Patient Type	Case vs. Control	2.880 (1.295, 6.407)	0.010*
	Medical Insurance			0.094
		Commercial vs. Public	1.590 (0.673, 3.754)	0.290
		Other vs. Public	7.637 (0.790, 73.864)	0.079
Patient Type + Presence of Medical Diagnosis^c^	Patient Type	Case vs. Control	3.779 (1.594, 8.958)	0.003
	Presence of Diagnosis	Yes vs. No	0.457 (0.106, 1.962)	0.292
Patient Type + Medication Use^a^	Patient Type	Case vs. Control	3.587 (1.595, 8.068)	0.002*
	Medication Use	Yes vs. No	0.143 (0.012, 1.673)	0.121
Patient Type + Preventive Visit Rate per year^a,d^	Patient Type	Case vs. Control	3.142 (1.370, 7.205)	0.007*
	Preventive visit	Each 1-visit increase	0.933 (0.683, 1.275)	0.663
Patient Type + Surface Number^a^	Patient Type	Case vs. Control	2.930 (1.388, 6.187)	0.005*
	Surface Number			0.019*
		2 vs. 1	2.129 (1.186, 3.822)	0.011*
		3 + vs. 1	2.376 (1.201, 4.702)	0.013*
		3 + vs. 2	1.116 (0.561, 2.221)	0.754

^a^The study cohort for this analysis included 21 positive Sjögren’s disease patients and 42 non-SD controls for a total of 63 patients. ^b^ The study cohort for this analysis included 20 cases and 41 controls. ^c^The study cohort for this analysis included 21 cases and 41 controls. ^d^The preventive visit rate per year was calculated by dividing the total number of preventive visits by the absolute value of the time between the index date and the patient’s first dental visit (of any type). Absolute times values less than one year were rounded to the nearest year. Asterisks (*) indicates statistical significance at the 5% level

**Table 5:** Univariable analysis for time to dental restoration failure by cases only (*n* = 102^a^)

Predictor	Comparison	Hazard Ratio (95% CI)	*p*-value
Age	Each 1-Year Change	1.008 (0.995, 1.022)	0.244
Sex	Female vs. Male	1.341 (0.795, 2.263)	0.272
Race			0.682
	Black vs. White	0.772 (0.412, 1.446)	0.419
	Unknown vs. White	1.029 (0.733, 1.446)	0.867
Dental Insurance	Other vs. Self-Pay	0.944 (0.654, 1.363)	0.759
Medical Insurance^b^			0.665
	Commercial vs. Public	1.013 (0.699, 1.467)	0.947
	Other vs. Public	1.718 (0.530, 5.568)	0.367
Presence of Medical Diagnosis	Yes vs. No	1.027 (0.253, 4.158)	0.971
Medication Use	Yes vs. No	1.687 (0.690, 4.124)	0.252
Preventive Visits Rate per year^c^	Each 1-visit increase	0.983 (0.918, 1.053)	0.623
Sjogren’s Status			0.426
	Negative vs. Positive	0.781 (0.538, 1.132)	0.192
	Uncertain vs. Positive	0.870 (0.565, 1.341)	0.529
Surface Number			0.213
	2 vs. 1	1.062 (0.729, 1.545)	0.755
	3 + vs. 1	1.446 (0.956, 2.188)	0.081

^**a**^ Univariate analysis includes 102 cases with 529 restorations, ^b^ includes 94 SD cases with 463 restorations. ^c^The preventive visit rate per year was calculated by dividing the total number of preventive visits by the absolute value of the time between the patient’s first dental visit and last dental visit (of any type). Absolute times values less than one year were rounded to the nearest year

Distribution of frequencies and percentages for selected medical conditions for cases and controls are provided in supplementary Table 3, Additional file 3. Analysis revealed significant differences between cases and controls for sialadenitis, rheumatoid arthritis, systemic lupus erythematosus, hypothyroidism, depressive disorder, myalgia and myositis/fibromyalgia, and anxiety and nervousness (*p* < 0.05). Depressive disorder was found in 57% of cases and was different from controls (*p* = 0.0012). Although hypertension was present in 71% of the cases, it was not different from non-SD controls (*p* > 0.4). Drug sub classes for the overall study cohort showed hydrocodone combinations representing at 73% followed by non-steroidal anti-inflammatory agents (NSAIDS) at 64%. For cases, hydrocodone combinations were at 74%, proton pump inhibitors at 71% and NSAIDs at 65%.

## Discussion

This analysis of 529 restorations among SD cases and 140 restorations among controls during the 15-year study time showed that direct restorations failed faster in patients with SD. Also, as reported among the general population [8, 16, 17, 54], restorations with 2 or more surfaces had a higher failure rate than one surface restorations. However, patient-related factors (age, race, socioeconomic status) including medical diagnosis and medications that potentially cause dry mouth did not influence restoration longevity. In addition, few existing studies have shown the influence of age, sex, and socio-economic status on restoration longevity [14, 54–56], which was not evident in this study similar to a practice based study [16]. A possible reason could be due to the matched case-control study design that resulted in low number of SD cases and corresponding control pairs that have at least one completed dental restoration even though the original cohort of patients with SD was large. Nevertheless, the high restoration failure rate among SD cases indicate that dentists may consider conservative dental treatments for patients with SD before initiating extensive dental treatment [57]. On the contrary fixed prosthetic crowns have shown longer survival time than composite restorations [34]. So, considering full coverage restorations such as a single unit crown for multi-surface carious involvement could be further investigated among patients with SD. In summary, dentists’ decisions regarding treatments along with tooth and patient related factors have a major influence on restoration longevity. In addition, ongoing assessment of caries risk factors and addressing them even after definitive treatment/s is critical to improve the prognosis of dental restorative treatments.

Caries preventive strategies might be effective with regular fluoride applications after placement of restorations or with fluoride-releasing filling material [58, 59]. In addition, the preventive management guidelines published by Zero et al. recommends salivary stimulants and non-fluoride remineralizing agents for caries prevention among SD patients [60]. However, these preventive measures are futile if they are implemented after occurrence of extensive dental tissue damage due to delay in diagnosing SD or failure to identify SD status during dental care. Therefore, early diagnosis of SD is necessary to prevent such irreversible damage to oral tissues and rest of the body. We have previously reported that oral health conditions such as caries may manifest even before SD diagnoses, necessitating dental treatments [61]. Therefore, dental clinicians may consider referring patients, especially women with a higher prevalence of caries and/or dry mouth to a rheumatologist or an oral pathologist to rule out SD. An early diagnosis could be achieved through co-ordination of medical and dental care and integration of EDR and EHR records can provide continuity of care for this patient population who experience significant dental disease burden that impacts their daily life.

It is a known fact that patients with SD maintain good oral hygiene [23, 28, 62]. Therefore, further studies are necessary to determine the role of underlying host factors in the rapid decline of restorations among cases. Our study findings also showed no significant difference in restoration failures among the three different case groups (positive, uncertain and negative cases) despite varying clinical findings. Further studies are also necessary to evaluate the effect of definitive treatments for SD such as immunosuppressants on dental conditions and dental treatment outcomes. Occurrence of dental restoration failures are often attributed to secondary caries, dislodgement, fracture, poor marginal integrity of the restoration material, oral hygiene status, and susceptibility to caries [7, 8, 13, 15, 19, 63, 64], which also need to be assessed.

In this study cohort, more than 70% of patients with SD have no dental insurance and self-paid for dental treatments. However, more than 90% had some form of medical insurance. This gap may be due to limited dental services coverage with the Healthy Indiana Plan (HIP) for ages 18 to 64 and no public dental insurance for those aged 65 and above [65]. About 45% of our patient cohort were over 60 years old, which might be the reason for the increased number of patients paying out of pocket. Moreover, advanced treatments, such as crowns, bridges, and dentures, are common among patients with SD [23, 66] and they incur substantial dental treatment costs because of extensive and multiple dental restorative procedures [31, 67, 68]. Therefore, expansion of dental coverage for patients with SD is necessary to improve timely access and management of their dental conditions.

The strength of our study relies on matching IUSD patients’ EDR with EHR information to identify patients with SD [38] and conduct this retrospective analysis. Yet, as with any research, this study has limitations. First, this study cohort included dental patients’ EDR data from one academic institution and therefore, any dental treatment received outside of this institution could be missing. For generalizability, similar studies should be conducted among multiple institutions. However, their EHR data has more coverage of their healthcare information because this information was retrieved from the state-wide health information exchange that receives healthcare information from more than 123 Indiana hospitals. Second, we could not study the patient, tooth- and restoration-level factors in detail because of the limited overall number of failures and missing data in some covariates. Although we employed a data set of 377 cases, analysis of cases by matching to their corresponding controls that have at least one complete direct restoration resulted in a smaller cohort consisting of 102 cases and 42 controls. This small sample size limited the possibilities to understand the natural history of carious outcomes related to tooth type (incisors, cuspids, premolars, and molars), location (maxillary or mandibular) and restoration material and extent. Third, we did not evaluate failure reasons because this information was present within the clinical notes and required additional processing to retrieve them. Other limitations in the study include not assessing the skill level of student and faculty providers performing restorative procedures. Also, we could not confirm the presence or absence of symptoms and diagnostic test results, which may be present in a different healthcare system that is inaccessible for research via the INPC-R database [38]. This is because healthcare systems have the right to decide what data can be used for research.

Despite these limitations, this study revealed a significant finding regarding the rapid decline of dental restorations among people with SD and the need to pay close attention even when planning direct dental restorations and monitor the various factors that may contribute to its survival through frequent follow-up. Another key finding is the need to diagnose SD early before irreversible damage occurs to various systems of the human body including the mouth. Therefore, further research is warranted that investigates the role of dental clinicians to refer high caries risk patients to a rheumatologist or an oral pathologist to rule out SD.

## Conclusion

The study results demonstrated that direct dental restorations declined rapidly with every subsequent replacement among SD case patients. It is a noteworthy finding that direct dental restorations among patients in the positive SD case group failed three times more often than among patients in the non-SD control group. In addition to emphasizing proper early diagnosis of SD, it is important to educate dental professionals to consider careful treatment planning especially when considering restoring teeth involving multiple surfaces. Preventive interventions such as fluoride applications, salivary stimulants, non-fluoride remineralizing agents have been recommended in patients with SD; however, future prospective clinical studies should investigate the effectiveness of preventive management after placement of restorations. Further studies are necessary to evaluate the role of dental clinicians in the early diagnosis of SD and referral of their patients especially female patients with a high caries risk.

### Electronic supplementary material

Below is the link to the electronic supplementary material.

Supplementary Material 1

Supplementary Material 2

Supplementary Material 3

Supplementary Material 4

## Data Availability

All relevant data are within the manuscript and its supporting information files. The raw data cannot be shared publicly because it contains protected health information such as date of birth and treatment dates. Additionally, these records are managed by the Regenstrief Institute, Inc. and the Indiana University School of Dentistry, Indianapolis, IN, and have imposed restrictions that prevent public sharing of this data set in accordance with the data sharing permissions assigned by patients and respective healthcare institutions. Interested researchers may contact Regenstrief Data Services with the request for data access via https://www.regenstrief.org/data-request/. The data set can be specified as Thankam Thyvalikakath’s R21 project, ‘Assessing the Oral Health and Dental Treatment Outcomes in Sjogren’s Syndrome’. Any request will require submission of the specific purpose of the data access for review and approval of the study cohort’s respective healthcare systems and the IU School of Dentistry privacy and compliance office before authorization for data access is granted.
