# Supplementary material for: Longevity of dental restorations in Sjogren’s disease patients using electronic dental and health record data
Source: BMC Oral Health. 2024 Feb 7;24:203. doi: 10.1186/s12903-024-03957-9 (PMC10848515; doi:10.1186/s12903-024-03957-9)
Supplement: Supplementary file 1 — Supplementary Material 1 [file 12903_2024_3957_MOESM1_ESM.docx]

**Supplementary Table 1: Initial direct dental restoration codes for amalgam and resin-based composites retrieved from electronic dental records (EDRs).**

| **Initial direct dental restoration codes for resin and amalgam** | | |
| --- | --- | --- |
| Category of dental procedures | CDT codes | Description of procedures |
| Restorative | D2100 | Amalgam Restorations |
|  | D2101 | IUSD-Amalgam polish^#^ |
|  | D2140 | Amalgam - one surface, primary or permanent |
|  | D2150 | Amalgam - two surfaces, primary or permanent |
|  | D2160 | Amalgam – three surfaces, primary or permanent |
|  | D2161 | Amalgam - four or more surfaces, primary or permanent |
|  | D2300 | Resin-Based Composite Restoration |
|  | D2330 | Resin-based composite-one surface, anterior |
|  | D2331 | Resin-based composite-two surfaces, anterior |
|  | D2332 | Resin-based composite- three surfaces, anterior |
|  | D2335 | Resin-based composite- four or more surfaces or involving incisal angle, anterior |
|  | D2390 | Resin-based composite crown, anterior |
|  | D2391 | Resin-based composite-one surface, posterior |
|  | D2392 | Resin-based composite-two surfaces, posterior |
|  | D2393 | Resin-based composite-three surfaces, posterior |
|  | D2394 | Resin-based composite-four or more surfaces, posterior. |
|  | VA | IUSD-Amalgam^#^ |
|  | D2649 | Resin-Based Composite |
|  | D2385 | Resin one surface, posterior permanent |
|  | D2386 | Resin two surfaces, posterior permanent |
|  | D2387 | Resin three or more surfaces, posterior permanent |
|  | D2388 | Resin four or more surfaces, posterior permanent |
|  | D2999 | Unspecified restorative procedures. |
|  | D2900 | Other Restorative Services |
| Material | E1002 | Amalgam Material |
|  | E1003 | Composite Material |

^#^Included internal institutional codes
