# Supplementary material for: Longevity of dental restorations in Sjogren’s disease patients using electronic dental and health record data
Source: BMC Oral Health. 2024 Feb 7;24:203. doi: 10.1186/s12903-024-03957-9 (PMC10848515; doi:10.1186/s12903-024-03957-9)
Supplement: Supplementary file 2 — Supplementary Material 2 [file 12903_2024_3957_MOESM2_ESM.docx]

**Supplementary Table 2: Failure codes and description of dental procedures subsequently present after placement of direct dental restoration among SDs and controls.**

| **Failure codes and procedures for direct dental restorations** | | |
| --- | --- | --- |
| Restorative |  |  |
|  | D2100 | Amalgam Restorations |
|  | D2101 | IUSD-Amalgam polish^#^ |
|  | D2140 | Amalgam - one surface |
|  | D2150 | Amalgam - two surfaces |
|  | D2160 | Amalgam - three surfaces, primary or permanent |
|  | D2161 | Amalgam – four or more surfaces, primary or permanent |
|  | D2300 | Resin-Based Composite Restoration |
|  | D2330 | Resin-based composite–one surface, anterior |
|  | D2331 | Resin-based composite–two surfaces, anterior. |
|  | D2332 | Resin-based composite–three surfaces, anterior |
|  | D2335 | Resin-based composite–four + surfaces, anterior. |
|  | D2390 | Resin-based composite crown, anterior |
|  | D2385 | Composite resin– one surface - posterior-permanent |
|  | D2386 | Composite resin– two surfaces - posterior-permanent |
|  | D2387 | Composite resin–three surfaces - posterior-permanent |
|  | D2388 | Composite resin–four or more surfaces - posterior-permanent |
|  | D2391 | Resin-based composite-one surface, posterior |
|  | D2392 | Resin-based composite-two surfaces, posterior |
|  | D2393 | Resin-based composite-three surfaces, posterior |
|  | D2394 | Resin-based composite- four or more surfaces, posterior |
|  | VA | IUSD-Amalgam^#^ |
|  | D2649 | Resin-Based Composite |
|  | D2400 | Gold Foil Restorations |
|  | D2410 | Gold foil - one surface |
|  | D2420 | Gold foil - two surfaces |
|  | D2430 | Gold foil - three surfaces |
|  | D2500 | Inlay/Onlay Restoration |
|  | D2501 | Metallic |
|  | D2510 | Inlay - metallic - one surface |
|  | D2520 | Inlay - metallic - two surfaces |
|  | D2530 | Inlay - metallic - three or more surfaces |
|  | D2542 | Onlay - metallic - two surfaces |
|  | D2543 | Onlay - metallic - three surfaces |
|  | D2544 | Onlay - metallic – four or more surfaces |
|  | D2600 | Porcelain/Ceramic |
|  | D2610 | Inlay - porcelain/ceramic - one surface |
|  | D2620 | Inlay - porcelain/ceramic - two surfaces |
|  | D2630 | Inlay - porcelain/ceramic – three or more surfaces |
|  | D2642 | Onlay – porcelain/ceramic - two surfaces |
|  | D2643 | Onlay - porcelain/ceramic - three surfaces |
|  | D2644 | Onlay - porcelain/ceramic- four or more surfaces |
|  | D2650 | Inlay - resin – one surface |
|  | D2651 | Inlay - resin–two surfaces |
|  | D2652 | Inlay - resin – three or more |
|  | D2662 | Onlay - resin – two surfaces |
|  | D2663 | Onlay - resin – three surfaces |
|  | D2664 | Onlay - resin – four or more surfaces |
|  | D2700 | Crowns - Single Restoration |
|  | D2710 | Crown - resin-based composite (indirect) |
|  | D2712 | Crown 3/4 resin indirect |
|  | D2720 | Crown - resin, high noble metal |
|  | D2721 | Crown - resin, predominantly base metal |
|  | D2722 | Crown - resin, noble metal |
|  | D2740 | Crown - porcelain/ceramic subs |
|  | D2750 | Crown - PFM^§^  high noble metal |
|  | D2751 | Crown - PFM^§^  predominantly base metal |
|  | D2752 | Crown - PFM^§^  noble metal |
|  | D2753 | Crown porcelain fused to titanium and titanium alloys |
|  | D2780 | Crown - 3/4 cast high noble metal |
|  | D2781 | Crown - 3/4 cast pred base metal |
|  | D2783 | Crown - 3/4 porcelain/ceramic |
|  | D2790 | Crown -Full cast high noble metal |
|  | D2791 | Crown -Full cast pred base metal |
|  | D2792 | Crown -Full cast noble metal |
|  | D2794 | Crown - titanium |
|  | D2799 | Provisional crown |
|  | D2900 | Other Restorative Services |
|  | D2910 | Re-cement or re-bond inlay, onlay, veneer, or partial coverage |
|  | D2915 | Re-cement or re-bond indirectly fabricated or prefabricated post and core |
|  | D2920 | Re-cement or re-bond crown |
|  | D2931 | Prefab SS crown - perm. tooth |
|  | D2932 | Prefab resin crown |
|  | D2933 | Prefab SS crown - resin window |
|  | D2940 | Protective restoration |
|  | D2950 | Core buildup - including pins |
|  | D2951 | Pin retention - per tooth |
|  | D2952 | Cast post and core |
|  | D2953 | Additional cast post - same tooth |
|  | D2954 | Prefabricated post and core in addition to crown |
|  | D2955 | Post removal |
|  | D2957 | Additional prefabricated post - same tooth |
|  | D2960 | Labial veneer, resin - direct |
|  | D2961 | Labial veneer, resin - indirect |
|  | D2962 | Labial veneer, porcelain - indirect |
|  | D2971 | Additional procedures to customize a crown to fit under an existing partial denture framework |
|  | D2980 | Crown repair necessitated by restorative material failure |
|  | D2981 | Inlay repair necessitated by restorative material failure |
|  | D2982 | Onlay repair necessitated by restorative material failure |
|  | D2983 | Veneer repair necessitated by restorative material failure |
|  | D2999 | Unspecified restorative procedure. |
|  | D6519 | Inlay/onlay -porcelain/ceramic |
|  | D6520 | Inlay - metallic–two surfaces |
|  | D6543 | Onlay – metallic–three surfaces |
|  | D6544 | Onlay - metallic–four + surfaces |
|  | D6612 | Onlay- base metal, two surfaces |
|  | D6615 | Onlay - cast noble metal, 3+ surfaces |
|  | D2782 | Crown-3/4 cast noble metal |
|  | D6782 | Crown- 3/4 cast pred. noble metal |
|  | D2970 | Temporary crown, fractured teeth |
| Material | E1002 | Amalgam Material |
|  | E1003 | Composite Material |
|  | E1001 | Gold Material |
|  | E1004 | Other Metal Material |
|  | E1005 | Porcelain Material |
|  | E1005P | Porcelain Fused to Metal |
|  | E1006 | Other Temporary Material |
|  | E1008 | Stainless Steel |
|  | E1010 | Gold Material on Implant |
|  | E1011 | Porcelain Material on Implant |
|  | E1015 | Post and Core |
|  | E2001 | Implant |
|  | E2100 | Pontic - Porcelain |
|  | E2101 | Pontic - Metal |
|  | E3001 | Endodontic Treatment |
|  | D3200 | Pulpotomy |
| Endodontics | D3000 | Pulp Capping |
|  | D3001 | Pulp Cap |
|  | D3110 | Pulp Cap, Direct |
|  | D3120 | Pulp Cap, Indirect |
|  | D3221 | Pulpal Debridement, Primary/Permanent |
|  | D3220 | Therapeutic Pulpotomy |
|  | D3229 | Pulpal Therapy |
|  | D3300 | Endodontic Therapy |
|  | D3310 | Endodontic Therapy - anterior |
|  | D3320 | Endodontic Therapy - bicuspid |
|  | D3330 | Endodontic Therapy - molar |
|  | D3331 | Endodontic therapy-Root canal obstruction-nonsurgical access |
|  | D3332 | Incomplete endodontic therapy; inoperable/fractured tooth; unrestorable or fractured tooth |
|  | D3333 | Endodontic - internal root repair of perforation defects |
|  | D3340 | Endodontic Retreatment |
|  | D3341 | Retreatment - Root Canal Therapy |
|  | D3346 | Retreatment of previous root canal therapy-Anterior |
|  | D3347 | Retreatment of previous root canal therapy -premolar |
|  | D3348 | Retreatment of previous root canal therapy -molar |
|  | D3355 | Pulpal Regeneration - initial visit |
|  | D3356 | Pulpal Regeneration - interim medication replacement |
|  | D3357 | Pulpal Regeneration - completion of treatment |
|  | D3400 | Apicoectomy/Periradicular Services |
|  | D3410 | Apicoectomy - anterior |
|  | D3421 | Apicoectomy - bicuspid(1st root) |
|  | D3425 | Apicoectomy - molar (1st root) |
|  | D3426 | Apicoectomy - additional roots |
|  | D3450 | Root amputation - per root |
|  | D3999 | Unspecified endo procedure |
| Surgery | D7000 | Extractions |
|  | D7140 | Extraction, erupted tooth |
|  | D7200 | Surgical Extractions |
|  | D7210 | Extraction, erupted tooth, req. bone removal, sectioning |
|  | D7250 | Removal of residual tooth roots |
|  | D7251 | Coronectomy - intentional partial tooth removal |
|  | D7310 | Alveoloplasty w/extraction > 3 teeth per quadrant |
|  | D7311 | Alveoloplasty including extraction: 1-3 teeth |
|  | D7320 | Alveoloplasty w/o extractions |
|  | D7321 | Alveoloplasty no extraction: 1-3 teeth |
|  | D7400 | Surgical Excision of Lesions |
|  | D7500 | Surgical Incision |
|  | D7510 | Incision/drainage, abscess-intraoral soft tissue |
|  | D7510E | IUSD-Incision/drainage, abscess-intraoral soft tissue^#^ |
|  | D7511 | Incision/drainage, abscess-intraoral soft tissue, complicated |
|  | D7520 | Incision/drainage, abscess-extraoral soft tissue |
|  | D7521 | Incision/drainage, abscess-extraoral soft tissue, complicated |
| Removable Prosthodontics | D5100 | Complete Dentures |
|  | D5110 | Complete denture - maxillary |
|  | D5120 | Complete denture - mandibular |
|  | D5130 | Immediate denture - maxillary |
|  | D5140 | Immediate denture - mandibular |
|  | D5200 | Partial Dentures |
|  | D5211 | Max. part denture - resin base |
|  | D5212 | Mandibular partial denture - resin base |
|  | D5213 | Max partial - cast metal frame |
|  | D5214 | Mandibular partial -cast metal frame |
|  | D5225 | Max RPD flexible base |
|  | D5226 | Mandibular RPD flexible base |
|  | D5227 | Immediate max partial denture flexible base |
|  | D5228 | Immediate mandibular partial dent flexible base |
|  | D5282 | Removable unilateral partial denture1 piece cast metal maxillary |
|  | D5283 | Removable unilateral partial denture 1piece cast metal mandibular |
|  | D5284 | Removable unilateral partial denture flexible base quadrant |
|  | D5286 | Removable unilateral partial denture resin quadrant |
|  | D5405 | Complete Denture |
|  | D5420 | Partial Denture |
|  | D5650 | Add tooth to existing partial |
|  | D5800 | Interim Prosthesis |
|  | D5810 | Interim complete denture – (Maxillary) |
|  | D5811 | Interim complete denture – (Mandibular) |
|  | D5820 | Interim partial denture - maxillary (flipper) |
|  | D5821 | Interim partial denture - mandibular (flipper) |
|  | D5840 | Other Removable Prosthodontic Services |
|  | D5863 | Overdenture - complete maxillary |
|  | D5864 | Overdenture - partial maxillary |
|  | D5865 | Overdenture - complete mandibular |
|  | D5866 | Overdenture - partial mandibular |
|  | D5875 | Modification of removable prosthesis following implant surgery |
|  | D5899 | Unspecified removable prosthodontic procedure by report |
|  | D5899A | IUSD-Cast base metal denture base^#^ |
|  | D5899B | IUSD-Cast high noble metal denture base^#^ |
|  | D5899C | IUSD-Cast gold occlusals per tooth^#^ |
|  | D6010 | Surgical placement of implant body, endosteal implant |
| Fixed prosthodontics | D6010B | IUSD-Surgical placement endosteal implant for predoc restoration^#^ |
|  | D6010P | IUSD-Surgical placement of implant body, palatal implant^#^ |
|  | D6011 | Second Stage Implant Surgery |
|  | D6011B | FP - Second Stage Implant Surgery |
|  | D6012 | Surgical placement of interim implant body for transitional prosthesis, endosteal implant |
|  | D6013 | Surgical placement of mini implant |
|  | D6040 | Surgical placement, eposteal implant |
|  | D6050 | Surgical placement, transosteal implant |
|  | D6050.1 | Implant Supported Prosthetics |
|  | D6065 | Implant - porcelain/ceramic crown |
|  | D6066 | Implant - PFM^§^, high noble metal |
|  | D6067 | Implant - metal crown, high noble metal |
|  | D6075 | Implant -supported retainer for ceramic FPD^@^ |
|  | D6076 | Implant -supported retainer for porcelain fused to metal PFM^§^  FPD^@^ |
|  | D6077 | Implant - supported retainer, for cast metal FPD^@^ |
|  | D6082 | Implant supported crown porcelain fused to base alloys |
|  | D6083 | Implant supported crown porcelain fused to noble alloys |
|  | D6084 | Implant supported crown porcelain fused to titanium |
|  | D6085 | Provisional Implant Crown |
|  | D6085B | Grad Periodontics Provisional Implant Crown |
|  | D6086 | Implant supported crown base alloys |
|  | D6087 | Implant supported crown noble alloys |
|  | D6088 | Implant supported crown titanium alloys |
|  | D6092 | Re-cement or re-bond implant supported crown |
|  | D6093 | Re-cement or re-bond implant/fixed partial denture FPD^@^ |
|  | D6199 | Unspecified implant procedure, by report |
|  | D6199A | Unspecified implant |
|  | D6199B | Unspecified implant |
|  | D6199C | Unspecified implant |
|  | D6200 | Prosthodontics–Fixed |
|  | D6201 | Fixed Partial Denture Pontics |
|  | D6205 | Pontic - indirect resin based |
|  | D6210 | Pontic - cast high noble metal |
|  | D6211 | Pontic - cast predominantly base metal |
|  | D6212 | Pontic - cast noble metal |
|  | D6212B | Pontic–Cast noble metal |
|  | D6214 | Pontic - titanium |
|  | D6240 | Pontic-porcelain fused to high noble |
|  | D6241 | Pontic-porcelain fused to base metal |
|  | D6242 | Pontic-porcelain fused to noble metal |
|  | D6242B | Pontic - Porcelain Fused–Noble Metal |
|  | D6243 | Pontic porcelain fused to titanium |
|  | D6245 | Pontic-porcelain/ceramic |
|  | D6245B | Pontic - Porcelain/Ceramic |
|  | D6250 | Pontic-resin, high noble metal |
|  | D6251 | Pontic-resin, predominantly base metal |
|  | D6252 | Pontic-resin with noble metal |
|  | D6253 | Provisional pontic |
|  | D6624 | Inlay - titanium |
|  | D6634 | Onlay - titanium |
|  | D6930 | Re-cement or re-bond bridge FPD^@^ |
|  | D6980 | Fixed partial denture FPD^@^ repair necessitated by restorative material failure |
|  | D6999 | Unspecified fixed prosthodontic procedure |
|  | D5215 | Upper partial high noble cast |
|  | D5216 | Lower partial high noble cast |
|  | D5281 | Unilateral Partial denture-1 piece cast metal |
| CPT codes | M10060 | Incision/drainage abscess, simple or single |
|  | M10061 | Incision/drainage, complex or multiple |
|  | M10180 | Incision/drainage, complex, posterior |
|  | M21501 | Incision/drainage, deep abscess |
|  | M40800 | Drainage of abscess, cyst, hematoma |
|  | M40805 | Removal, embedded fb, complicated |
|  | M40810 | Excision of lesion of mucosa/s |
|  | M40814 | Excision lesion of mucosa/submucosa |
|  | M40820 | Destruction of lesion or scar |
|  | M41000 | Intraoral incision/drainage of abscess, cyst,  or hematoma of tongue or floor of mouth; lingual |
|  | M41005 | Intraoral incision/drainage of abscess, cyst,  or hematoma of tongue or floor of mouth; sublingual, superficial |
|  | M41006 | Sublingual deep supramylohyoid |
|  | M41007 | Intraoral incision/drainage of abscess, cyst, or hematoma of tongue or floor of mouth; sublingual, superficial |
|  | M41008 | Intraoral incision/drainage of abscess, cyst, or hematoma of tongue or floor of mouth; sublingual, deep, supramylohyoid |
|  | M41009 | Intraoral incision/drainage of abscess, cyst,  or hematoma of tongue or floor of mouth; masticator space |
|  | M41015 | Extraoral i/d abscess, sublingual |
|  | M41016 | Extraoral Incision & Drainage |
|  | M41017 | Extraoral incision/drainage floor of mouth |
|  | M41018 | Extraoral incision/drainage abscess/cyst |
|  | M41899 | Unspecified procedures., dentoalveolar |
|  | M41899O | Root Canal Anterior (D3310) |
|  | M41899P | Root Canal Bicuspid (D3320) |
|  | M41899Q | Root Canal Molar (D3330) |
|  | M41899S | Extraction Erupted/Exposed Teeth (D7140) |
|  | M41899Y | Ext Root Tip Surgical (D7250) |
|  | M42300 | Drainage abscess, parotid, simple |
|  | M42305 | Drainage abscess, parotid, complications |
|  | M42310 | Drain abscess submaxillary/sublingual intraoral |
|  | M42320 | Drain abscess, submaxillary, extraoral |
|  | M42720 | Incision/drain abscess; intraoral |
|  | M42725 | Incision drainage, external approach |

^#^ Included internal institutional codes; ^@^FPD- Fixed partial denture; ^§^ PFM- Porcelain fused to metal
