# Supplementary material for: Longevity of dental restorations in Sjogren’s disease patients using electronic dental and health record data
Source: BMC Oral Health. 2024 Feb 7;24:203. doi: 10.1186/s12903-024-03957-9 (PMC10848515; doi:10.1186/s12903-024-03957-9)
Supplement: Supplementary file 3 — Supplementary Material 3 [file 12903_2024_3957_MOESM3_ESM.docx]

**Supplementary Table 3: Diagnostic counts for selected medical conditions from electronic health records (EHRs) that potentially cause dry mouth.**

| **Medical conditions** | **Overall N=144** | | **Case N=102** | | **Control N=42** | | **p-value** |
| --- | --- | --- | --- | --- | --- | --- | --- |
|  | N | (%) | N | (%) | N | (%) |  |
| **Sialadenitis** |  |  |  |  |  |  | 0.0376* |
| No | 133 | (93.0) | 92 | (90.2) | 41 | (100.0) |  |
| Yes | 10 | (7.0) | 10 | (9.8) |  |  |  |
| **Sialolithiasis** |  |  |  |  |  |  | > 0.99 |
| No | 141 | (98.6) | 100 | (98.0) | 41 | (100.0) |  |
| Yes | 2 | (1.4) | 2 | (2.0) |  |  |  |
| **Rheumatoid Arthritis** |  |  |  |  |  |  | 0.0037* |
| No | 114 | (79.7) | 75 | (73.5) | 39 | (95.1) |  |
| Yes | 29 | (20.3) | 27 | (26.5) | 2 | (4.9) |  |
| **Systemic Lupus Erythematosus** |  |  |  |  |  |  | 0.0207* |
| No | 120 | (83.9) | 81 | (79.4) | 39 | (95.1) |  |
| Yes | 23 | (16.1) | 21 | (20.6) | 2 | (4.9) |  |
| **Crohn’s Disease** |  |  |  |  |  |  | > 0.99 |
| No | 142 | (99.3) | 101 | (99.0) | 41 | (100.0) |  |
| Yes | 1 | (0.7) | 1 | (1.0) |  |  |  |
| **Ulcerative Colitis** |  |  |  |  |  |  | 0.49 |
| No | 141 | (98.6) | 101 | (99.0) | 40 | (97.6) |  |
| Yes | 2 | (1.4) | 1 | (1.0) | 1 | (2.4) |  |
| **Hypothyroidism** |  |  |  |  |  |  | 0.0436* |
| No | 86 | (60.1) | 56 | (54.9) | 30 | (73.2) |  |
| Yes | 57 | (39.9) | 46 | (45.1) | 11 | (26.8) |  |
| **Diabetes** |  |  |  |  |  |  | 0.88 |
| No | 92 | (64.3) | 66 | (64.7) | 26 | (63.4) |  |
| Yes | 51 | (35.7) | 36 | (35.3) | 15 | (36.6) |  |
| **Anemia** |  |  |  |  |  |  | 0.12 |
| No | 76 | (53.1) | 50 | (49.0) | 26 | (63.4) |  |
| Yes | 67 | (46.9) | 52 | (51.0) | 15 | (36.6) |  |
| **Cushing’s Syndrome** |  |  |  |  |  |  |  |
| No | 143 | (100.0) | 102 | (100.0) | 41 | (100.0) |  |
| **Depressive Disorder** |  |  |  |  |  |  | 0.0012* |
| No | 74 | (51.7) | 44 | (43.1) | 30 | (73.2) |  |
| Yes | 69 | (48.3) | 58 | (56.9) | 11 | (26.8) |  |
| **Hypertension** |  |  |  |  |  |  | 0.40 |
| No | 45 | (31.5) | 30 | (29.4) | 15 | (36.6) |  |
| Yes | 98 | (68.5) | 72 | (70.6) | 26 | (63.4) |  |
| **Myalgia and Myositis/Fibromyalgia** |  |  |  |  |  |  | 0.0023* |
| No | 99 | (69.2) | 63 | (61.8) | 36 | (87.8) |  |
| Yes | 44 | (30.8) | 39 | (38.2) | 5 | (12.2) |  |
| **Circumscribed Scleroderma** |  |  |  |  |  |  | > 0.99 |
| No | 142 | (99.3) | 101 | (99.0) | 41 | (100.0) |  |
| Yes | 1 | (0.7) | 1 | (1.0) |  |  |  |
| **Parkinson's Disease** |  |  |  |  |  |  |  |
| No | 143 | (100.0) | 102 | (100.0) | 41 | (100.0) |  |
| **Alzheimer's Disease** |  |  |  |  |  |  | > 0.99 |
| No | 139 | (97.2) | 99 | (97.1) | 40 | (97.6) |  |
| Yes | 4 | (2.8) | 3 | (2.9) | 1 | (2.4) |  |
| **Systemic Sclerosis** |  |  |  |  |  |  | 0.19 |
| No | 136 | (95.1) | 95 | (93.1) | 41 | (100.0) |  |
| Yes | 7 | (4.9) | 7 | (6.9) |  |  |  |
| **Mixed Connective Tissue Disease** |  |  |  |  |  |  | > 0.99 |
| No | 141 | (98.6) | 100 | (98.0) | 41 | (100.0) |  |
| Yes | 2 | (1.4) | 2 | (2.0) |  |  |  |
| **Bell's Palsy** |  |  |  |  |  |  | > 0.99 |
| No | 142 | (99.3) | 101 | 99.0) | 41 | (100.0) |  |
| Yes | 1 | (0.7) | 1 | (1.0) |  |  |  |
| **Stress** |  |  |  |  |  |  | 0.11 |
| No | 130 | (90.9) | 90 | (88.2) | 40 | (97.6) |  |
| Yes | 13 | (9.1) | 12 | (11.8) | 1 | (2.4) |  |
| **Atrophic Gastritis** |  |  |  |  |  |  | > 0.99 |
| No | 142 | (99.3) | 101 | (99.0) | 41 | (100.0) |  |
| Yes | 1 | (0.7) | 1 | (1.0) |  |  |  |
| **Chronic Fatigue Syndrome** |  |  |  |  |  |  | 0.41 |
| No | 136 | (95.1) | 98 | (96.1) | 38 | (92.7) |  |
| Yes | 7 | (4.9) | 4 | (3.9) | 3 | (7.3) |  |
| **Renal Diseases** |  |  |  |  |  |  | 0.58 |
| No | 139 | (97.2) | 98 | (96.1) | 41 | (100.0) |  |
| Yes | 4 | (2.8) | 4 | (3.9) |  |  |  |
| **Hyperparathyroid** |  |  |  |  |  |  |  |
| No | 143 | (100.0) | 102 | (100.0) | 41 | (100.0) |  |
| **Anxiety and Nervousness** |  |  |  |  |  |  | 0.0039* |
| No | 105 | (73.4) | 68 | (66.7) | 37 | (90.2) |  |
| Yes | 38 | (26.6) | 34 | (33.3) | 4 | (9.8) |  |
| **Autoimmune Thyroid Condition** |  |  |  |  |  |  | > 0.99 |
| No | 138 | (96.5) | 98 | (96.1) | 40 | (97.6) |  |
| Yes | 5 | (3.5) | 4 | (3.9) | 1 | (2.4) |  |
| **Actinomycosis** |  |  |  |  |  |  |  |
| No | 143 | (100.0) | 102 | (100.0) | 41 | (100.0) |  |
| **Cytomegalovirus** |  |  |  |  |  |  |  |
| No | 143 | (100.0) | 102 | (100.0) | 41 | (100.0) |  |
| **Hemochromatosis** |  |  |  |  |  |  |  |
| No | 143 | (100.0) | 102 | (100.0) | 41 | (100.0) |  |

*Asterisks represent significance at 5% level.
