# Supplementary material for: Longevity of dental restorations in Sjogren’s disease patients using electronic dental and health record data
Source: BMC Oral Health. 2024 Feb 7;24:203. doi: 10.1186/s12903-024-03957-9 (PMC10848515; doi:10.1186/s12903-024-03957-9)
Supplement: Supplementary file 4 — Supplementary Material 4 [file 12903_2024_3957_MOESM4_ESM.docx]

**Supplementary Table 4: Drug group, class, and subclass of commonly prescribed medications among SD cases** **and non-SD controls.**

|  | | | **Overall**  **(N=144)** | | **Case**  **(N=102)** | | **Control**  **(N=42)** | |
| --- | --- | --- | --- | --- | --- | --- | --- | --- |
| **Drug Group** | **Drug Class** | **Drug Sub Class** | **N** | **(%)** | **N** | **(%)** | **N** | **(%)** |
| **Analgesics - Anti-Inflammatory** | Nonsteroidal Anti-Inflammatory Agents (NSAIDs) | Cyclooxygenase 2 (COX-2) Inhibitors | 19 | (13.2) | 13 | (12.7) | 6 | (14.3) |
|  |  | Nonsteroidal Anti-inflammatory Agent Combinations | 2 | (1.4) | 1 | (1.0) | 1 | (2.4) |
|  |  | Nonsteroidal Anti-inflammatory Agents (NSAIDs) | 92 | (63.9) | 66 | (64.7) | 26 | (61.9) |
|  | Pyrimidine Synthesis Inhibitors | Pyrimidine Synthesis Inhibitors | 5 | (3.5) | 5 | (4.9) | 0 | (0.0) |
|  | Soluble Tumor Necrosis Factor Receptor Agents | Soluble Tumor Necrosis Factor Receptor Agents | 4 | (2.8) | 4 | (3.9) | 0 | (0.0) |
| **Analgesics - Opioid** | Opioid Agonists | Opioid Agonists | 59 | (41.0) | 49 | (48.0) | 10 | (23.8) |
|  | Opioid Combinations | Codeine Combinations | 30 | (20.8) | 26 | (25.5) | 4 | (9.5) |
|  |  | Hydrocodone Combinations | 105 | (72.9) | 75 | (73.5) | 30 | (71.4) |
|  |  | Meperidine Combinations | 1 | (0.7) | 1 | (1.0) | 0 | (0.0) |
|  |  | Opioid Combinations | 43 | (29.9) | 31 | (30.4) | 12 | (28.6) |
|  |  | Propoxyphene Combinations | 40 | (27.8) | 33 | (32.4) | 7 | (16.7) |
|  |  | Tramadol Combinations | 3 | (2.1) | 2 | (2.0) | 1 | (2.4) |
|  | Opioid Partial Agonists | Opioid Partial Agonists | 2 | (1.4) | 2 | (2.0) | 0 | (0.0) |
| **Antianxiety Agents** | Antianxiety Agents - Miscellaneous | Antianxiety Agents - Miscellaneous | 36 | (25.0) | 27 | (26.5) | 9 | (21.4) |
|  | Benzodiazepines | Benzodiazepines | 51 | (35.4) | 43 | (42.2) | 8 | (19.0) |
| **Antiasthmatics and Bronchodilators** | Anti-Inflammatory Agents | Anti-Inflammatory Agents | 1 | (0.7) | 1 | (1.0) | 0 | (0.0) |
|  | Bronchodilators - Anticholinergics | Bronchodilators - Anticholinergics | 6 | (4.2) | 6 | (5.9) | 0 | (0.0) |
|  | Leukotriene Modulators | Leukotriene Receptor Antagonists | 15 | (10.4) | 15 | (14.7) | 0 | (0.0) |
|  | Steroid Inhalants | Steroid Inhalants | 19 | (13.2) | 19 | (18.6) | 0 | (0.0) |
|  | Sympathomimetics | Adrenergic Combinations | 22 | (15.3) | 22 | (21.6) | 0 | (0.0) |
|  |  | Beta Adrenergics | 43 | (29.9) | 43 | (42.2) | 0 | (0.0) |
|  |  | Mixed Adrenergics | 0 | (0.0) | 0 | (0.0) | 0 | (0.0) |
|  | Xanthines | Xanthines | 4 | (2.8) | 4 | (3.9) | 0 | (0.0) |
| **Anticonvulsants** | Anticonvulsants - Benzodiazepines | Anticonvulsants - Benzodiazepines | 15 | (10.4) | 13 | (12.7) | 2 | (4.8) |
|  | Anticonvulsants - Miscellaneous | Anticonvulsants - Miscellaneous | 58 | (40.3) | 47 | (46.1) | 11 | (26.2) |
|  | Hydantoins | Hydantoins | 2 | (1.4) | 2 | (2.0) | 0 | (0.0) |
|  | Valproic Acid | Valproic Acid | 4 | (2.8) | 3 | (2.9) | 1 | (2.4) |
| **Antidepressants** | Alpha-2 Receptor Antagonists (Tetracyclics) | Alpha-2 Receptor Antagonists (Tetracyclics) | 10 | (6.9) | 10 | (9.8) | 0 | (0.0) |
|  | Antidepressants - Miscellaneous | Antidepressants - Miscellaneous | 21 | (14.6) | 16 | (15.7) | 5 | (11.9) |
|  | Monoamine Oxidase Inhibitors (MAOIs) | Monoamine Oxidase Inhibitors (MAOIs) | 0 | (0.0) | 0 | (0.0) | 0 | (0.0) |
|  | Selective Serotonin Reuptake Inhibitors (SSRIs) | Selective Serotonin Reuptake Inhibitors (SSRIs) | 63 | (43.8) | 51 | (50.0) | 12 | (28.6) |
|  | Serotonin Modulators | Serotonin Modulators | 31 | (21.5) | 26 | (25.5) | 5 | (11.9) |
|  | Serotonin-Norepinephrine Reuptake Inhibitors (SNRI) | Serotonin-Norepinephrine Reuptake Inhibitors (SNRI) | 34 | (23.6) | 29 | (28.4) | 5 | (11.9) |
|  | Tricyclic Agents | Tricyclic Agents | 30 | (20.8) | 26 | 25.5) | 4 | 9.5) |
| **Antiemetics** | Antiemetics - Anticholinergic | Antiemetics - Anticholinergic | 24 | (16.7) | 17 | (16.7) | 7 | (16.7) |
| **Antihypertensives** | ACE Inhibitors | ACE Inhibitors | 49 | (34.0) | 34 | (33.3) | 15 | (35.7) |
|  | Angiotensin II Receptor Antagonists | Angiotensin II Receptor Antagonists | 30 | (20.8) | 17 | (16.7) | 13 | (31.0) |
|  | Antiadrenergic Antihypertensives | Antiadrenergics - Centrally Acting | 15 | (10.4) | 13 | (12.7) | 2 | (4.8) |
|  |  | Antiadrenergics - Peripherally Acting | 8 | (5.6) | 6 | (5.9) | 2 | (4.8) |
|  | Antihypertensive Combinations | ACE Inhibitor & Calcium Channel Blocker Combination | 3 | (2.1) | 1 | (1.0) | 2 | (4.8) |
|  |  | ACE Inhibitor & Calcium Channel Blocker Combinations | 0 | (0.0) | 0 | (0.0) | 0 | (0.0) |
|  |  | ACE Inhibitors & Thiazide/Thiazide-Like | 8 | (5.6) | 5 | (4.9) | 3 | (7.1) |
|  |  | Angiotensin II Receptor Antagonist & Thiazide/Thiazide | 7 | (4.9) | 3 | (2.9) | 4 | (9.5) |
|  |  | Beta Blocker & Diuretic Combinations | 1 | (0.7) | 1 | (1.0) | 0 | (0.0) |
|  |  | Direct Renin Inhibitors & Thiazide/Thiazide-Like | 0 | (0.0) | 0 | (0.0) | 0 | (0.0) |
|  | Direct Renin Inhibitors | Direct Renin Inhibitors | 0 | (0.0) | 0 | (0.0) | 0 | (0.0) |
|  | Selective Aldosterone Receptor Antagonists (SARAs) | Selective Aldosterone Receptor Antagonists (SARAs) | 1 | (0.7) | 1 | (1.0) | 0 | (0.0) |
|  | Vasodilators | Vasodilators | 6 | (4.2) | 5 | (4.9) | 1 | (2.4) |
| **Corticosteroids** | Mineralocorticoids | Mineralocorticoids | 1 | (0.7) | 1 | (1.0) | 0 | (0.0) |
| **Cough/Cold/ Allergy** | Cough/Cold/Allergy Combinations | Decongestant-Antihistamine-Anticholinergic | 5 | (3.5) | 2 | (2.0) | 3 | (7.1) |
| **Diuretics** | Carbonic Anhydrase Inhibitors | Carbonic Anhydrase Inhibitors | 1 | (0.7) | 0 | (0.0) | 1 | (2.4) |
|  | Diuretic Combinations | Diuretic Combinations | 18 | (12.5) | 13 | (12.7) | 5 | (11.9) |
|  | Loop Diuretics | Loop Diuretics | 30 | (20.8) | 24 | (23.5) | 6 | (14.3) |
|  | Potassium Sparing Diuretics | Potassium Sparing Diuretics | 5 | (3.5) | 5 | (4.9) | 0 | (0.0) |
|  | Thiazides and Thiazide-Like Diuretics | Thiazides and Thiazide-Like Diuretics | 44 | (30.6) | 32 | (31.4) | 12 | (28.6) |
| **Fluoroquinolones** | Fluoroquinolones | Fluoroquinolones | 77 | (53.5) | 55 | (53.9) | 22 | (52.4) |
| **Genitourinary Agents - Miscellaneous** | Prostatic Hypertrophy Agents | Alpha 1-Adrenoceptor Antagonists | 5 | (3.5) | 5 | (4.9) | 0 | (0.0) |
| **Musculoskeletal Therapy Agents** | Central Muscle Relaxants | Central Muscle Relaxants | 60 | (41.7) | 44 | (43.1) | 16 | (38.1) |
| **Nasal Agents - Systemic and Topical** | Nasal Agent Combinations | Antihistamine-Steroid | 1 | (0.7) | 1 | (1.0) | 0 | (0.0) |
|  | Nasal Antiallergy | Nasal Antihistamines | 7 | (4.9) | 7 | (6.9) | 0 | (0.0) |
|  | Nasal Anticholinergics | Nasal Anticholinergics | 0 | (0.0) | 0 | (0.0) | 0 | (0.0) |
|  | Nasal Steroids | Nasal Steroids | 49 | (34.0) | 49 | (48.0) | 0 | (0.0) |
|  | Sympathomimetic Decongestants | Systemic Decongestants | 9 | (6.3) | 9 | (8.8) | 0 | (0.0) |
|  |  | Topical Decongestants | 2 | (1.4) | 2 | (2.0) | 0 | (0.0) |
| **Ulcer drugs/ Antispasmodics/ Anticholinergics** | Antispasmodics | Quaternary Anticholinergics | 0 | (0.0) | 0 | (0.0) | 0 | (0.0) |
|  | H-2 Antagonists | H-2 Antagonists | 43 | (29.9) | 43 | (42.2) | 0 | (0.0) |
|  | Proton Pump Inhibitors | Proton Pump Inhibitors | 72 | (50.0) | 72 | (70.6) | 0 | (0.0) |
